# Psychological assessment in edentulous patients before and after complete denture

**DOI:** 10.6026/973206300210504

**Published:** 2025-03-31

**Authors:** Rahul A Razdan, Rajeev Srivastava, Trapti Jaiswal, Monica Garg, Harsh Chansoria, Atul Kala

**Affiliations:** 1 Department of Prosthodontics and Crown & Bridge, Index Institute of Dental Sciences, Indore, Madhya Pradesh, India; 2 Department of Prosthodontics and Crown & Bridge, Sri Aurobindo College of Dentistry, Indore, Madhya Pradesh, India; 3 Department of Prosthodontics and Crown & Bridge, Government College of Dentistry, Indore, Madhya Pradesh, India; 4 Department of Oral & Maxillofacial Surgery, Index Institute of Dental Sciences, Indore, Madhya Pradesh, India

**Keywords:** Complete dentures, psychological assessment, edentulous patients, confidence, mastication, pronunciation, quality of life

## Abstract

The psychological well-being of edentulous patients before and after receiving complete dentures, focusing on parameters such as confidence in smiling, ability to eat and pronunciation is of interest. Data shows substantial improvements in all assessed parameters are found. Confidence in smiling and eating showed the highest levels of improvement, while pronunciation difficulties significantly decreased. Thus, the transformative impact of complete dentures on enhancing both oral function and overall psychosocial well-being is highlighted.

## Background:

Edentulism, the complete loss of natural teeth, poses a significant challenge to a patient's quality of life, impacting essential functions such as mastication, speech and overall facial aesthetics [1]. Beyond the physical impairments, edentulism often results in psychological distress, reducing self-confidence and social interaction due to perceived inadequacies in appearance, difficulties in eating and problems with speech articulation [2, 3]. These factors collectively affect an individual's emotional well-being and social functionality, emphasizing the need for effective prosthodontic interventions [4]. Complete dentures remain a widely utilized and accessible solution for the restoration of oral function and esthetics in edentulous patients [5, 6].

While the primary goal of complete dentures is to restore physiological function, their impact on psychological parameters is equally vital [7, 8]. Enhancing a patient's confidence to smile, eat comfortably and speak clearly can substantially improve their overall quality of life [9]. Therefore, it is of interest to evaluate and compare the psychological well-being of edentulous patients before and after receiving complete dentures, focusing on critical aspects such as confidence in smiling, ability to eat and difficulties in pronunciation.

## Materials and Methods:

This interventional study was conducted at the Department of Prosthodontics and Crown & Bridge, Index Institute of Dental Sciences, Indore (Madhya Pradesh) and involving completely edentulous patients. The inclusion criteria comprised individuals without muscular or temporo-mandibular joint disorders or psychiatric conditions. The sample size of 52 participants was determined using G*Power 3.1.9.7 software, with an effect size of 0.5, α probability error of 0.05, and power of 0.95, resulting in a critical χ^2^ value of 3.841. Eligible patients provided informed consent, followed by a detailed case history and pre-treatment psychological assessments focusing on confidence in smiling, ability to eat and difficulties in pronunciation. Complete dentures were fabricated and post-treatment evaluations of the same parameters were conducted one week after denture insertion. Data were systematically recorded in an Excel sheet and analyzed using SPSS (version 25.0). Normality was assessed via the Kolmogorov-Smirnov test, with descriptive statistics applied for general data distribution. Categorical variables were analyzed using the Chi-square test, while continuous variables were compared using One-way ANOVA or the Kruskal-Wallis test, with a significance threshold set at p < 0.05 (Table 1).

## Results:

The study included a total of 52 participants, with an age distribution ranging from 40 to 60 years and a mean age of 52.4 years. The gender distribution comprised 30 males and 22 females. Psychological assessments conducted before and after the insertion of complete dentures revealed significant improvements across all evaluated parameters. (Annexure 1- questionnaire) Confidence in smiling improved markedly, with mean scores increasing from 2.4 ± 0.8 before treatment to 4.6 ± 0.5 after denture insertion (p < 0.001). Similarly, confidence in eating demonstrated a substantial enhancement, rising from a pre-treatment mean of 2.2 ± 0.7 to a post-treatment mean of 4.5 ± 0.6 (p < 0.001).

Pronunciation difficulties, a notable concern among edentulous patients, significantly decreased following denture placement, with mean scores dropping from 3.8 ± 0.9 before treatment to 1.6 ± 0.4 after treatment (p < 0.001). Statistical analysis confirmed the improvements were highly significant (p < 0.001), indicating the profound impact of complete denture treatment on the psychological well-being of patients. Among the parameters assessed, confidence in smiling and eating exhibited the most pronounced positive changes, while difficulties in pronunciation were significantly alleviated, highlighting the comprehensive benefits of complete dentures in restoring both functional and psychosocial aspects of oral health (Table 2) (Figure 1 - see PDF).

## Demographics:

[1] Age distribution: 40-60 years (mean age: 52.4 years)

[2] Gender distribution: 30 males, 22 females

## Statistical analysis:

[1] A significant improvement was observed in all psychological parameters post-treatment (p < 0.001).

[2] Confidence in smiling and eating showed the most considerable improvement.

[3] Pronunciation difficulties significantly reduced after denture insertion.

## Discussion:

Edentulism, the complete loss of natural teeth, profoundly affects individuals both functionally and psychologically [10]. Beyond impairing essential activities such as eating and speaking, it often leads to diminished self-esteem and social withdrawal due to altered facial aesthetics and compromised oral functions [11]. Complete dentures have long been employed to restore oral functionality and appearance in edentulous patients. While their efficacy in improving masticatory function is well-documented, their impact on psychological well-being warrants thorough exploration [12, 13]. Our study demonstrated significant enhancements in psychological parameters following complete denture treatment. Specifically, patients exhibited increased confidence in smiling and eating, alongside a notable reduction in pronunciation difficulties. These findings align with existing literature emphasizing the psychological benefits of complete dentures [14]. For instance, a study published in the Journal of International Society of Preventive and Community Dentistry reported that both male and female edentulous patients experienced improved psychological comfort during mastication after receiving complete dentures. Although no statistical difference was observed between genders, a numerical predominance of psychological satisfaction was noted among female patients [15].

Furthermore, research in the Journal of Clinical and Diagnostic Research highlighted that the psychological attitude of denture wearers significantly influences their adaptation and acceptance of the prosthesis. Philosophical and exacting patients demonstrated better adaptation to dentures compared to hysterical and indifferent individuals, underscoring the importance of considering psychological factors in prosthodontic treatment [14]. Additionally, a study in the Journal of Prosthodontics concluded that personality traits affect patients' acceptance of complete dentures. Patients with high neuroticism scores were less satisfied with their dentures, indicating that psychological profiles can influence treatment outcomes [16]. These studies corroborate our findings, suggesting that complete dentures not only restore oral function but also significantly enhance psychological well-being. By improving confidence in smiling and eating, and reducing pronunciation difficulties, complete dentures contribute to better social interactions and overall quality of life for edentulous patients.

In conclusion, our study adds to the growing body of evidence that complete denture treatment offers substantial psychological benefits, reinforcing the need for a holistic approach in prosthodontic rehabilitation that addresses both functional and emotional aspects of edentulism. Soboleva *et al.* Patient satisfaction with complete dentures was not influenced by age, sex or the degree of resorption. Instead, overall satisfaction with both maxillary and mandibular dentures was primarily linked to comfort and aesthetics. Additionally, patient comfort was closely associated with the stability of the mandibular denture [17]. Goyal *et al.* conducted a study evaluating the satisfaction of edentulous patients with complete dentures using a patient denture assessment. Despite advancements in prosthodontics, emotional well-being and patient satisfaction with denture fabrication have received limited attention. The study involved patients from the Department of Prosthodontics at Sharad Pawar Dental College, who completed a questionnaire assessing their satisfaction with the delivered prosthesis. The results were analyzed based on patient responses, providing insights into their overall experience with complete dentures [18].

## Conclusion:

Complete dentures positively impact the psychological parameters of edentulous patients, significantly improving confidence and reducing difficulties in speech. Thus, the role of prosthodontic interventions in enhancing quality of life is highlighted.

## Figures and Tables

**Table 1:** Structured questionnaire for the study

**Domain**	**Question**	**Response Options**
Confidence in Smile	Before receiving complete dentures, how confident do you feel about your smile?	A. Very Confident B. Somewhat Confident C. Neutral D. Not Confident at All
	After receiving complete dentures, how confident do you feel about your smile?	A. Very Confident B. Somewhat Confident C. Neutral D. Not Confident at All
	Comparing your smile confidence before and after dentures, how would you rate the change?	A. Improved Significantly B. Improved Somewhat C. No Significant Change D. Declined
Confidence in Eating	Before getting complete dentures, how confident are you in your ability to eat comfortably?	A. Very Confident B. Somewhat Confident C. Neutral D. Not Confident at All
	After receiving complete dentures, how confident are you in your ability to eat comfortably?	A. Very Confident B. Somewhat Confident C. Neutral D. Not Confident at All
	Comparing you is eating confidence before and after dentures, how would you rate the change?	A. Improved Significantly B. Improved Somewhat C. No Significant Change D. Declined
Pronunciation Difficulties	Before having complete dentures, do you experience difficulties in pronunciation?	A. Rarely or Never B. Occasionally C. Frequently D. Always
	After getting complete dentures, do you still experience difficulties in pronunciation?	A. Rarely or Never B. Occasionally C. Frequently D. Always
	Comparing your pronunciation difficulties before and after dentures, how would you rate the change?	A. Improved Significantly B. Improved Somewhat C. No Significant Change D. Declined

**Table 2:** Psychological assessments

**Parameter**	**Before Denture (Mean ± Standard Deviation)**	**After Denture (Mean ± Standard Deviation)**	**P - value**
Confidence in smiling	2.4 ± 0.8	4.6 ± 0.5	<0.001
Confidence in eating	2.2 ± 0.7	4.5 ± 0.6	<0.001
Pronunciation difficulties	3.8 ± 0.9	1.6 ± 0.4	<0.001

## References

[1] Emami E (2013). Int J Dent..

[2] Peltzer K (2014). Int J Environ Res Public Health..

[3] Vadavadagi S.V (2015). J Int Oral Health..

[4] Bhochhibhoya A (2022). The Journal of Prosthetic Dentistry..

[5] Martins A.M.C (2021). Dent Res J (Isfahan)..

[6] Damian J.L, Saponaro P.C (2019). Dental Clinics of North America..

[7] Bansod A.V (2022). Cureus..

[8] Chiddarwar R (2024). Cureus..

[9] Shingane S (2024). J Pharm Bioallied Sci..

[10] Wayler A.H, Chauncey H.H (1983). J Prosthet Dent..

[11] Fisher J (2023). NAM Perspect..

[12] Carlsson G.E, Omar R (2010). J Oral Rehabil..

[13] Turkyilmaz I (2010). Gerodontology..

[14] Dhaded S (2022). J Indian Prosthodont Soc..

[15] Seenivasan M.K (2019). Cureus..

[16] Fouda S.M (2014). Int J Dent..

[17] Soboleva U, Rogovska I (2022). Medicina (Kaunas)..

[18] Goyal P (2021). Journal of Pharmaceutical Research International..

